# Use of modified composite index of anthropometric failure and MUAC-for-age to assess prevalence of malnutrition among school-age children and adolescents involved in the school feeding program in Addis Ababa, Ethiopia

**DOI:** 10.1186/s40795-021-00471-x

**Published:** 2021-11-26

**Authors:** Zelalem Destaw, Eshetu Wencheko, Samuel Zemenfeskidus, Yohannes Challa, Melkamu Tiruneh, Meti Tamrat Fite, Dilu Shaleka, Mogessie Ashenafi

**Affiliations:** 1 Center for Food Security Studies, College of Development Studies, Addis Ababa University, Addis Ababa, Ethiopia; 2 Department of Statistics, College of Natural and Computational Sciences, Addis Ababa University, Addis Ababa, Ethiopia; 3 Addis Ababa Health Bureau, Addis Ababa, Ethiopia; 4 Addis Ababa Education Bureau, Addis Ababa, Ethiopia; 5 College of Development Studies, Addis Ababa University, Addis Ababa, Ethiopia

**Keywords:** Adolescents, BMI-for-age, mCIAF, MUAC-for-age, Nutritional status, School feeding, School-age children

## Abstract

**Background:**

Malnutrition hampers educational performance of schoolchildren coming from low-income families. School feeding program was, thus, launched in public primary schools in Addis Ababa very recently. It is, thus, important to measure the initial nutritional status of participating students to see the effect of the program on their nutritional wellbeing.

**Methods:**

The first-round survey was made at the initiation of the program. A multi-stage stratified sampling from 50 schools located in the ten sub cities of Addis Ababa yielded 4500 children and adolescents of ages five to 19 years. Data was collected on age, height, weight and MUAC of the schoolchildren. Nutritional status was evaluated using conventional anthropometric indicators, modified Composite Index of Anthropometric Failure (mCIAF), and MUAC-for-age. Receiver Operating Characteristic (ROC) curve was used to examine classification of malnourishment by MUAC-for-age versus BMI-for-age and mCIAF versus MUAC-for-age. Multilevel mixed effects model was applied to investigate variations in the prevalence of malnutrition across sub cities.

**Findings:**

The area under the ROC curves (AUC) for MUAC-for-age against BMI-for-age z-scores was 0.68 and that of mCIAF against MUAC-for-age was 0.70, respectively, indicating an overall better classification of malnourishment. Mixed effects model showed significant variations in nutritional status of schoolchildren across sub cities. Conventional measures showed that prevalence of stunting, thinness, or underweight among the sample children and adolescents was 23.4, 18.4, and 16.5%, respectively. Assessment by mCIAF, instead, showed a higher prevalence of overall malnutrition (43.4%). MUAC-for-age indicated an acute malnutrition measurement of 33.4%. Significant differences (*p* < 0.0001) in nutritional status were seen between boys and girls, and among age groups as measured by mCIAF.

**Interpretation:**

Conventional measures of nutritional status undermined level of malnutrition. Instead, mCIAF and MUAC-for-age gave higher estimates of the magnitude of the existing prevalence of malnutrition among the school children and adolescents.

Summary **What is already known?**

- Assessment of nutritional status of school-age children and adolescents using conventional measures such as height-for-age, weight-for-age, weight-for-height, and BMI-for-age might underestimate the prevalence to malnutrition since each indicated only a single nutritional outcome.
- There were recent developments that recommended use of a Composite Index of Anthropometric Failure (CIAF) to assess aggregate level of malnutrition and MUAC-for-age for school-age children and adolescents to evaluate acute malnutrition.

**What are the new findings?**

- Initial nutritional status of school-age children and adolescents involved in the Addis Ababa school feeding program is determined using a modified Composite Index of Anthropometric Failure (mCIAF), the conventional measures, and MUAC-for-age.
- Despite considerable burden of malnutrition that existed among the school children and adolescents, the individual conventional measures undermined the problem.

**What do the new findings imply?**

- Use of mCIAF to assess nutritional status of school-age children and adolescents would preferably provide comprehensive measure of the magnitude of the aggregate anthropometric failure than the individual conventional indices.
- Assessment of severe and moderate acute malnutrition condition of school-age children and adolescents using sex-specific MUAC-for-age may be used to identify those seeking medication or nutritional intervention.
- Evidence herein might fill existing gap in nutritional condition of school-age children and adolescents among the sub cities of Addis Ababa.

## Introduction

School-age children and adolescents are characterized by a rapid and intense physiological, mental and social development with high nutritional requirement to maintain such changes [1, 2]. Poor nutrition may expose them to long-term consequences or risks of bad nutritional and health outcomes both during adolescence and adulthood [2].

In order to avoid risks of unhealthy outcomes and to maintain health and normal growth, children should get sufficient nutritious food that meets their dietary requirement. However, worldwide, large number of children suffer from burdens of undernutrition attributed to risk factors such as poverty, conflicts, shocks, disaster, poor dietary intake and disease [3].

In Ethiopia, at least two out of five under-five children suffer from chronic malnutrition [4]. Adolescent girls and boys of age 15–19 years old in Ethiopia are prone to chronic energy deficiency and, in Addis Ababa, 15% of under-five children are stunted [5]. The initiative of the Addis Ababa school feeding program was intended to alleviate hunger among poor school children, improve their attendance, minimize rates of dropout and repeaters and, ultimately, enhance educational performances of students [6].

The Addis Ababa City Administration launched a wider school feeding program in February 2019, though, previously, school meal provisions had been underway in some schools by charitable societies and other stakeholders [6]. During the duration of the current study, the program initiated by the City government involved about 51, 689 school children and adolescents, in 221 public primary schools distributed over ten sub cities of Addis Ababa [6].

There is no available information on the nutritional status of school-age children and adolescents involved in the Addis Ababa school feeding program. WHO uses growth references based on estimated Z-scores for height-for-age, weight-for-age, weight-for-height, and BMI-for-age for children and adolescents of ages between five and 19 years, which help to evaluate nutritional outcomes such as stunting, underweight, wasting, and thinness, respectively [7]. However, each of the standard indices of stunting, underweight, wasting, and thinness may underestimate the prevalence to malnutrition since each indicates only a single nutritional outcome [8]. There are various methodological changes that better estimate nutritional statuses of school-aged children and adolescents [7–12].

Since recently, an aggregate measure of prevalence of undernutrition, namely Composite Index of Anthropometric Failure (CIAF) has been suggested to assess the overall prevalence of undernourishment [8].

The CIAF is constructed from seven components or subgroups of anthropometric failure [8]. These are (*i*) stunting only; (*ii*) wasting only; (*iii*) underweight only; (*iv*) stunting and underweight; (*v*) wasting and underweight; (*vi*) stunting, wasting and underweight; and (*vii*) no anthropometric failure. The CIAF method has been applied and validated by some studies to evaluate nutritional status of preschool children [13]. However, both the above mentioned studies [8, 13] did not take overweight into consideration.

CIAF was implemented to evaluate anthropometric failure of pre-school and school-age children by including an additional category, namely excess weight (obesity and overweight) [9], which is measured by the weight-for-age index. This raised the number of indicators that determined CIAF from seven to nine. The extra two groups were overweight, and overweight combined with stunting [9, 12].

Weight-for-age can also be used as a measure of underweight. However, it does not distinguish between height and body mass for ages older than 10 years [10]. Use of weight-for-age would be misleading for children and adolescents older than 10 years as pubertal growth spurt may result in apparent excess weight (by weight-for-age) while, in fact, they are just tall [10]. Thus, considering weight-for-age in hitherto CIAF computations can be applied only for children of age five to 9 years [10]. Moreover, there is no WHO reference to assess weight-for-height Z-score for school-age children and adolescents [7, 10]. We, thus, modified the composite index by including BMI-for-age, which is preferred over weight-for-height Z-score for school-age and adolescents as it assesses changes in weight and height by accounting for age [14].

Another growth reference, MUAC, conventionally was not specific to sex and age. Recently, a new growth reference for sex-specific MUAC-for-age, has also been proposed for children and adolescents of ages five to 19 years concurrent with the WHO growth references [11].

The recently introduced indices, such as MUAC-for-age and CIAF, to the hitherto implemented three WHO standard indices (weight-for-age, height-for-age, and BMI-for-age), would provide a comprehensive information about the nutritional status of school-age children and adolescents.

Several studies from Addis Ababa showed that the nutritional status of school-age children was far from desirable [15–17]. As student academic performance and school attendance are believed to be influenced by nutrition, it was appropriate to assess the nutritional status of schoolchildren at the initiation of the school feeding program to evaluate its benefits. Thus, this study aimed at assessing the nutritional status of school-aged children and adolescents involved in the initial phase of the school feeding program in public primary schools in Addis Ababa using mCIAF and sex-dependent MUAC-for-age, in addition to the conventional anthropometric indices.

## Methods

The data for this study was collected in June, 2019, following the initiation of the school feeding program (SFP) on February 11, 2019 by the Addis Ababa Education Bureau (AAEB) [6]. The program involved 51,689 schoolchildren whose families were identified poor by local committee consisting of the smallest administration unit (called *Woreda)*, school teachers and directors, in 221 public primary schools distributed over all (ten) sub cities of Addis Ababa. The study focused on school-aged children and adolescents of age between five and 19 years [3],, who participated in SFP in sampled primary schools in Addis Ababa.

According to the education system in Ethiopia, primary education consists of the initial eight grades, comprised of primary 1st cycle (grades 1–4), and primary 2nd cycle (grades 5–8) [18, 19].

Sample size determination was based on a repeated measurement study design, and the current work was based on the baseline data, using the formula: [20, 21]

$$ \boldsymbol{n}={\left({\boldsymbol{z}}_{\left(\mathbf{1}-\frac{\boldsymbol{\alpha}}{\mathbf{2}}\right)}+{\boldsymbol{z}}_{\boldsymbol{\beta}}\right)}^{\mathbf{2}}\times \frac{\left[\mathbf{1}+\left(\boldsymbol{r}-\mathbf{1}\right)\boldsymbol{\rho} \right]}{\boldsymbol{r}{\boldsymbol{\varepsilon}}^{\mathbf{2}}} $$

Where, ***r*** is the number of time points of measurement; ***ρ*** and ***ε*** are the correlation of the repeated measures and effect size of intervention, respectively, assumed based on Cohen [20], ***β*** is the desired probability of rejection of null hypothesis; and ***α*** is the desired level of significance. Based on this, a sample size of 90 targeted schoolchildren per school was determined at an effect size of the intervention (0.3), 0.5 correlation, 5% level of significance, 80% power, design effect of 1.5, and adjusting for 90% response and attrition rate.

Addis Ababa is comprised of ten sub cities (called *kifle-Ketemas)*, each with an average population size of 300,727 (195,273 to 546,219) [22]. According to a population projection until 2037, children and adolescents of age five–19 currently constituted 22% of the city population [23]. Each sub city is further divided into 10 to 15 *Woredas*. Based on a multi-stage cluster sampling design, five districts were randomly selected from each sub-city, followed by random selection of one primary school from each selected district. A total of 50 primary schools and 4500 schoolchildren were, thus, considered for the study. Schoolchildren at each grade level were listed and randomly selected based on proportion of their sexes.

Anthropometric measurements were taken by qualified health professionals before lunch was served to students and within 3 days of start of measurement. Weight and height were measured by trained health professionals following WHO child growth Standards [24]. They were given training on measurement procedures to avoid variability. Weight was measured using a UNISCALE (a digital scale made by UNICEF) after removing outer clothings. Height was measured using a stadiometer (height board) mounted at a right angle between a level floor and against a wall. The schoolchild removed shoes and stood upright for height measurement.

The nutritional status of the school-aged children and adolescents was analyzed based on the anthropometric measurements and the status was compared by age groups and sex. Z-scores of Height-for-Age, Weight-for-Age, and BMI-for-age were computed using WHO Anthro Plus software [10]. Prevalence rates of stunting, thinness, and overweight were reported based on the WHO Anthro Plus estimates. Although growth reference to compute weight-for-age Z-score was available for children younger than 10 years old, WHO Anthro Plus software calculated prevalence of underweight (low weight-for-age Z-score) based on the total sample size that included 10 to 19 year old children and adolescents. This underestimated the prevalence of underweight in under 10 year old children.

Thus, the correct prevalence rate for underweight was separately calculated for five-to-nine years old children only. The classification of level of malnutrition was determined based on cutoffs set in WHO Growth Reference [10].

In our modified CIAF, weight-for-age was computed only for children of age five to nine^.7^ Similarly, since weight-for-height was recommended only for under-five children, BMI-for-age was used for schoolchildren who were 5 years or older [10]. To construct the mCIAF for school-age children and adolescents, we used thinness (low BMI-for-age) instead of wasting (low weight-for-height) and excess weight, in our case, was for high BMI-for-age and not for high weight-for-height.

The data was separately computed for age groups of five to nine, 10–14 and 15–19 years as the computation and interpretations, according to WHO AnthroPlus, were technically and theoretically different across the age groups.

The modified CIAF (mCIAF) assigned school-age children and adolescents into “No failure” groups, when they attained normal growth status by all the three conventional indices, namely stunting, thinness, underweight/excess weight. Children and adolescents who failed to attain a normal growth in, at least, one of the standard indices were categorized as “Failure”. The nine groups of mCIAF, as modified from previous works [8, 9, 12], are given in Table 1.

**Table 1:** mCIAF categories adapted for children and adolescents of age group 5–19 years

Categ-ories	Description	Stunting	Underweight	Thinness	Overweight
A	Without anthropometric failure	No	No	No	No
B	Thinness only	No	No	Yes	No
C*	Thinness and Underweight	No	Yes	Yes	No
D	Stunting, Thinness, and Underweight	Yes	Yes	Yes	No
E*	Stunting and Underweight	Yes	Yes	No	No
F	Stunting only	Yes	No	No	No
G	Overweight	No	No	No	Yes
H	Stunting and excess weight (overweight and obese)	Yes	No	No	Yes
Y*	Underweight only	No	Yes	No	No

Adatpted from others with modification for school-age children and adolescents [9, 12]

*Only for schoolchildren of age group 5–9 years [10]

Thus, proportion of Anthropometric Failure is the sum of the percentages of each failure groups indicated in Table 1, except group A, and can be calculated as:

$$ mCIAF=\left(1-A\right)\%. $$

The sex-specific MUAC-for-age Z-scores, as a measure of acute malnutrition of severe (Z-score < − 3) and moderate (Z-score < − 2) types, was calculated based on the reference data provided by Mramba et al. [11] using the formula:

$$ \mathrm{Z}-\mathrm{score}=\frac{{\left(\frac{y}{M}\right)}^L-1}{\mathrm{L}\times \mathrm{S}} $$

where ***y*** is observed child’s MUAC measurement, M is median MUAC of the reference group, S is coefficient of variation of the reference group, and L is the skewness of the reference group.

The level of the correlation between MUAC-for-age Z-score and Z-scores of conventional anthropometric measures was examined using Pearson’s product-moment correlation. ROC Curve analysis was used to examine performance of the MUAC-for-age to correctly classify nutritional status against BMI-for-age. Similarly, the performance of mCIAF in correctly classifying anthropometric failure was compared against MUAC-for-age. Further, covariates-adjusted receiver operating characteristic (ROC) regression was used to fit ROC curve under sex and age categories.

Outliers were excluded from the analysis. Chi-square test of association was used to examine differences in nutritional outcomes between boys and girls and among sub cities.

Multilevel mixed effects regression model was fitted in order to evaluate the level of variations in the measures of nutritional status across levels of sub cities.

### Ethics approval and consent to participate

All methods were carried out in accordance with the Declaration of Helsinki. Permission was obtained from the Addis Ababa Education Bureau, the responsible body for the school feeding program. The study was approved by the Institutional Review Board (IRB) of the Addis Ababa Public Health Research and Emergency Management Directorate, Addis Ababa Health Bureau with Ref. No. 36552/227. Informed consent form was signed and obtained from parents or guardians to take anthropometric measurements of their under-18 year old children and adolescents. Adolescents older than 18 years gave informed oral consent to be measured anthropometrically. Anthropometric measurements were administered by qualified health professionals. Confidentiality of information and anonymity of participants were maintained.

## Results

Data from a total of 4500 children and adolescents, consisting of 46.2% girls and 53.8% boys, was analyzed after outliers were excluded. An increase in height, weight and MUAC was observed with increase in age in both sexes. Lower height and MUAC was observed than those in WHO reference in age groups 10–14 and 15–19 (Table 2).

**Table 2:** Median Height (cm), Weight (Kg) and MUAC (mm) measurements of children and adolescents

Age (years)	Sex	N	Height		Weight		MUAC	
			Observed (IQR)	Ref^a^.	Observed (IQR)	Ref^a^.	Observed (IQR)	Ref^c^.
5–9	Girls	760	126 (10)	120.9	24 (5.5)	22.8	16.6 (2)	18.3
	Boys	845	125.7 (9.8)	121.6	23 (5.5)	23.1	16.5 (1.8)	18
10–14	Girls	1252	142 (14.5)	150.2	34 (11)	NA^b^	19 (3.5)	22.8
	Boys	1508	139.7 (14.1)	149.8	31 (8.3)	NA	18 (2.5)	22.6
15–19	Girls	67	153 (9.8)	162.5	48 (11)	NA	22 (3.7)	25.8
	Boys	68	158.6 (11.2)	173.3	45 (7.7)	NA	20.8 (3)	28.1

*IQR* Inter Quartile Range

^a^Median computed from 2007 WHO reference [7]

^b^NA, not available

^c^Median computed from the reference group used by Mramba et al. [11]

Although estimation of each component of mCIAF among our study subjects ranged from 0.8 to 13.7%, overall mCIAF indicated that a much higher proportion (43.4%) of the school-age children and adolescents were malnourished (Table 3). Composite anthropometric failure was observed in 39.7% of all girls, with the highest proportion observed in age group 10 to 14 (44.1%). Similarly, 46.6% of all boys manifested a composite anthropometric failure, and the highest proportion (51.5%) was seen in the age group 15 to 19.

**Table 3:** Composite index anthropometric failure among school-age children and adolescents involved in school feeding program, Addis Ababa, Public Primary Schools

Modified CIAF			Girls				Boys				Overall
			5–19 years	5–9 years	10–14 years	15–19 years	5–19 years	5–9 years	10–14 years	15–19 years	5–19 years
			n				n				N
			(%)	%	%	%	%	%	%	%	%
A	Without anthropometric failure		1253 (60.3)	67.2	55.9	62.7	1294 (53.5)	59.5	50.3	48.5	2547 (56.6)
B	Thinness only		196 (9.4)	6.8	11.3	4.5	306 (12.6)	7.2	15.7	13.2	502 (11.2)
C	Thinness and Underweight		48 (2.3)	6.3	–	–	74 (3.1)	8.8	–	–	122 (2.7)
D	Stunting, Thinness, and Underweight		68 (3.3)	1.5	4.4	3.0	136 (5.6)	2.3	7.3	10.3	204 (4.5)
E	Stunting and Underweight		22 (1.1)	2.9	–	–	57 (2.4)	6.8	–	–	79 (1.8)
F	Stunting only		271 (13.1)	4.3	18.3	13.4	343 (14.2)	3.9	19.6	22.1	614 (13.7)
G	Excess weight (overweight and obese)		122 (5.9)	7.1	4.9	10.5	118 (4.9)	6.3	4.2	2.9	240 (5.3)
H	Stunting and excess weight		88 (4.2)	2.4	5.3	6.0	68 (2.8)	2.4	3.1	2.9	156 (3.5)
Y	Underweight only		11 (0.5)	1.5	–	–	25 (1)	3.0	–	–	36 (0.8)
**Total N (%)**			2079 (100)	760 (100)	1252 (100)	67 (100)	2421 (100)	845 (100)	1508 (100)	68 (100)	4500 (100)
	mCIAF	N	826 (39.7)	249	552	25	1127 (46.6)	342	750	35	1953 (43.4)
		%		32.8	44.1	37.3		40.5	49.7	51.5	

Table 4 shows the summary of the proportion and standard deviations (SD) of school-aged and adolescents that were under aggregate anthropometric failure, namely stunted, underweight, and thin. It also shows if the indicators of nutritional status were significantly varying across sex at each age groups of five to nine, 10–14, and 15–19 years.

**Table 4:** Prevalence of malnutrition by age and sex among school-age children and adolescents involved in school feeding program, Addis Ababa, Primary Schools

Indicator	Age group (years)	Girls		Boys		Both		Sex difference *χ*^2^-value
		n	%(SD)	n	%(SD)	n	%(SD)	
Stunting	5–9	760	11.1 (1.3)	845	15.3 (1.2)	1605	13.3 (1.2)	6.1*
	10–14	1252	27.9 (1.3)	1508	29.9 (1.2)	2760	29.0 (1.2)	1.3
	15–19	67	22.4 (1.2)	68	35.3 (1.1)	135	28.9 (1.2)	2.7^**.**^
	Total	2079	21.6 (1.3)	2421	24.9 (1.3)	4500	23.4 (1.3)	7.1*
Underweight^a^	5–9	760	12.0 (1.02)	845	20.6 (1.1)	1605	16.5 (1.1)	25.7***
Thinness	5–9	760	14.6 (1.4)	845	18.2 (1.4)	1605	16.5 (1.4)	4.3
	10–14	1252	15.7 (1.4)	1508	22.9 (1.4)	2760	19.6 (1.4)	27.5***
	15–19	67	7.5 (1.2)	68	23.5 (1.3)	135	15.6 (1.4)	9.1*
	Total	2079	15.0 (1.4)	2421	21.3 (1.4)	4500	18.4 (1.3)	34.0***
Excess weight	5–9	760	9.4 (1.4)	845	8.7 (1.4)	1605	9.0 (1.4)	4.3
	10–14	1252	10.1 (1.4)	1508	7.2 (1.4)	2760	8.6 (1.4)	27.5***
	15–19	67	16.4 (1.2)	68	6.0 (1.3)	135	11.1 (1.3)	9.0*
	Total	2079	10.1 (1.4)	2421	7.6 (1.4)	4500	8.7 (1.4)	34.0***
mCIAF^§^	5–9	760	32.8 (3)	845	40.5 (2.7)	1605	36.5 (1.9)	10.2**
	10–14	1252	44 (2.1)	1508	49.7 (1.8)	2760	47.2 (1.3)	8.8**
	15–19	67	37.3 (9.6)	68	51.5 (8.6)	135	44.4 (2.2)	2.7^**.**^
	Total	2079	39.7 (1.7)	2421	46.6 (1.5)	4500	43.4 (1.1)	20.5***

Sex difference statistically significant at ***p < 0.0001, **p < 0.01, *p < 0.05, ^**.**^*p* < 0.1

^a^Weight-for-age reference data are not available beyond age 10 [10]

The modified aggregate measure of malnutrition, mCIAF, showed that an overall high prevalence of malnutrition among school-aged children and adolescents involved in school feeding program. In contrast, the conventional indices of stunting, thinness and underweight each undermined the prevalence rates and could not describe the overall prevalence of malnutrition independently (Table 4).

Prevalence of overall acute malnutrition among our study subjects ranged between 24.1 and 48.9%, with the lowest proportion seen among the age group five to 9 years (Table 5). Moderate acute malnutrition was observed in all age groups of both sexes at a rate of 23 to 25%, though the rate was significantly lower (*p* < 0.0001) in age group five to 9 years (18%). Severe acute malnutrition, on the other hand, was notably the highest (26%) among age group 15 to 19 years. All differences among age groups were statistically significant (p < 0.0001).

**Table 5:** Acute malnutrition status of school-age children and adolescents based on sex-specific MUAC-for-age

Age group	Girls		Boys		Both sexes			Overall (Acute malnutrition)
	MAM (%)	SAM (%)	MAM, (%)	SAM, (%)	MAM, (%)	SAM, (%)	N	
5–9	107 (14.1)	23 (3.0)	181 (21.4)	76 (8.9)	288 (17.9)	99 (6.2)	1605	24.1
10–14	258 (20.6)	89 (7.1)	435 (28.9)	267 (17.7)	693 (25.1)	356 (12.9)	2760	38.0
15–19	10 (14.9)	8 (11.9)	21 (30.9)	27 (39.7)	31 (22.9)	35 (25.9)	135	48.9
Total	375 (18.0)	120 (5.8)	637 (26.3)	370 (15.3)	1012 (22.5)	490 (10.9)	4500	33.4

NB: Both sex and age group differences are statistically significant at p < 0.0001

*SAM* Severe Acute Malnutrition with Z-score < −3SD, *MAM* Moderate Acute Malnutrition with Z-score (−3SD and, −2SD] [11]

Girls in all age groups showed lower (p < 0.0001) prevalence of moderate acute malnutrition than did boys (Table 5). Similarly, severe acute malnutrition had higher prevalence in boys of all age groups. It is worth noting that prevalence of moderate acute malnutrition in girls and moderate and severe acute malnutrition in boys increased with age. Similarly, prevalence of overall acute malnutrition increased along with increase in age. All differences among both sexes were statistically significant (*p* < 0.0001).

MUAC-for-age was strongly correlated with weight-for-age and BMI-for-age (r = 0.64 and 0.54, respectively). Its correlation with height-for-age, however, was moderate (r = 0.39) (Table 6).

**Table 6:** Correlation between Z-score measures of anthropometry with that of MUAC-for-age

Measure	N	Correlation	95% Confidence Interval	
Height-for-age	4500	0.38	0.36	0.41
Weight-for-age	1605	0.64	0.61	0.67
BMI-for-age	4500	0.52	0.50	0.54

The area under the receiver operating characteristic curves (AUC) for MUAC-for-age (classified as acute malnourished or normal) against BMI-for-age z-scores (classified as thin or normal) was 0.68 and that of mCIAF against MUAC-for-age was 0.70, respectively (Table 7).

**Table 7:** Area under the ROC curves for MUAC-for-age against BMI-for-age and that of mCIAF against MUAC-for-age

Reference	Classifier	ROC^a^ Area	SE^b^	Asymptotic Normal [95% CI^c^]	
BMI-for-age	MUAC-for-age	0.68	0.009	0.66	0.70
MUAC-for-age	mCIAF	0.70	0.008	0.69	0.72

^a^*ROC* Receiver operating characteristic

^b^*SE* Standard Error

^c^*CI* Confidence Interval

**Fig. 1:**
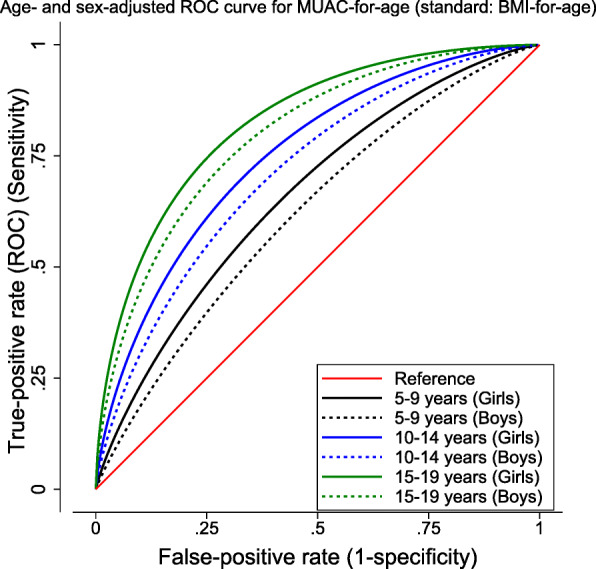
ROC curve for MUAC-for-age by age and sex with reference to BMI-for-age

**Fig. 2:**
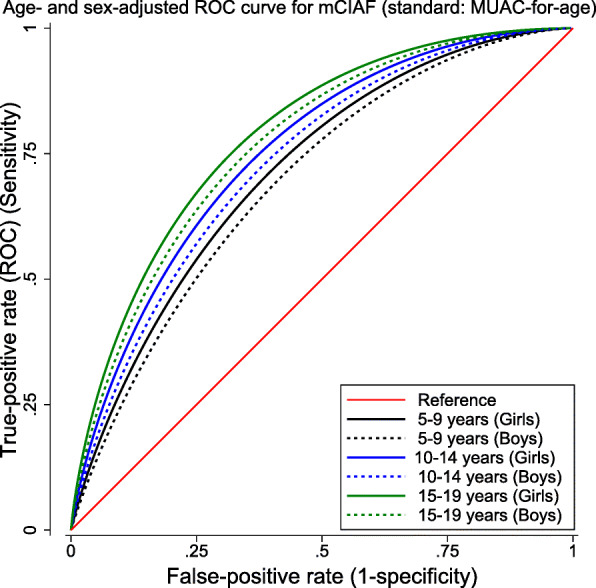
ROC curve for mCIAF by age and sex with reference to MUAC-for-age

This showed that the MUAC-for-age classification of acute malnutrition performed closely fair with BMI-for-age (Table 7, Fig. 1); and fair classification of malnourishment was captured by mCIAF as compared with MUAC-for-age (Table 7, Fig. 2). The area under the ROC curve (AUC) for boys and older ages was wider than that for girls and younger ages, respectively, after sex- and age group-adjusted ROC regression was fitted (Figs. 1 and 2). Considerably more agreement was observed in detecting malnourishment in early- and late-adolescents in both figures. More specifically, the curves fitted at each age group for mCIAF and MUAC-for-age showing closer gap between curves. These indicated an evident overall better classification of malnutrition.

MUAC-for-age measurements also showed that 30 and 20% of our study subjects, who suffered from anthropometric failures, also manifested moderate (Z-score < − 2) and severe (Z-score < − 3) malnutrition [11], respectively (Table 8). Prevalence of both moderate and severe malnutrition was significantly higher in boys than in girls in all age groups (*p* < 0.0001). The difference was much wider, however, in prevalence of severe malnutrition, particularly in the age group 15–19 years.

**Table 8:** Prevalence of SAM and MAM among school-age and adolescent suffering from anthropometric failure

Age group	Sex	MAM, %(n)	SAM, %(n)	Normal, %(n)	Total, N	Sex Difference *χ*^2^-value
5–9	Girls	50 (20.1)	18 (7.3)	181 (73.0)	249	15.2***
	Boys	93 (27.2)	51 (14.9)	198 (58.0)	342	
10–14	Girls	166 (30.1)	78 (14.1)	308 (55.8)	552	61.8***
	Boys	264 (35.2)	217 (28.9)	269 (35.9)	750	
15–19	Girls	5 (20)	4 (16)	16 (64)	25	12.3**
	Boys	7 (20)	20 (57)	8 (23)	35	
5–19	Girls	221 (26.8)	100 (12.1)	505 (61.1)	826	82.5***
	Boys	364 (32.3)	288 (25.6)	475 (42.2)	1127	
	Both	585 (30)	388 (19.9)	980 (50.2)	1953	

***p < 0.0001, **p < 0.01

*SAM* Severe Acute Malnutrition with Z-score < −3SD, *MAM* Moderate Acute Malnutrition with Z-score (−3SD and, −2SD] [11]

**Fig. 3:**
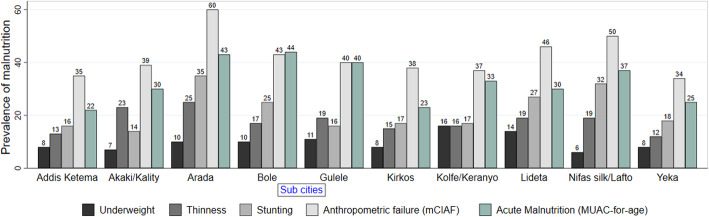
Prevalence of undernutrition among school-age and adolescents involved in school feeding program in each sub cities

Prevalence of malnutrition, as measured by mCIAF, ranged between 35 and 62% among our study subjects in all sub cities in Addis Ababa. It was the highest in *Arada* sub city and lowest in *Yeka* sub city. The prevalence of acute malnutrition also ranged between 23 and 44% among the sub cities. Prevalence of stunting followed that of acute malnutrition was considerably high in *Arada* (37%), *Nifas Silk/Lafto* (33%) and *Lideta* (30%) sub cities. The lowest prevalence (≤16%) of underweight was seen in six of the ten sub cities (Fig. 3).

## Discussion

Although the conventional indices of stunting, thinness or underweight each undermined the prevalence of malnutrition, overall mCIAF showed much higher proportion of malnourished schoolchildren. Beneficiaries of school feeding programs, during this study, were students who came from low-income households and could not get enough food that could sustain them through the school day [6]. Thus, the high prevalence of malnutrition, as indicated by mCIAF, should be expected among the observed population of schoolchildren targeted for school feeding program.

The observed variations in nutritional status across sex at each age group, as observed in both the individual and composite index measures, were significant (*p* < 0.0001) and indicative of differences in prevalence of malnutrition among boys and girls of school-aged children and adolescents. A similar observation was noted in Eastern and North Eastern Ethiopia where boys were found more affected than girls [25].

Studies on nutritional status of school-age children and adolescent in different parts of Ethiopia showed varying proportion of prevalence of malnutrition [25, 26]. However, these studies were based only on the conventional anthropometric indicators, and did not consider a composite measure of anthropometric failure. As observed in our study, use of the composite measure would result in a higher rate of prevalence than shown by the conventional measures.

A pooled estimate of prevalence in Ethiopia showed that 21.3, 18.2, and 17.7% of school-age children and adolescents were stunted, underweight or thin, respectively [17], and the pooled prevalence of stunting in Addis Ababa was 22% [17]. These figures were similar to those obtained by conventional methods in our study. The Global Nutrition Report (GNR) estimates of underweight in adolescent in Ethiopia (2016) was 35.6 and 21.9% in boys and girls, respectively [27]. However, the definition that GNR used for underweight was a Z-score of more than one standard deviation below the median BMI-for-age of the WHO growth reference [27]. The equivalent estimate of the current study would, thus, be 58.5% boys and 41.5% girls, which was higher than that reported by GNR [27].

However, inconsistent findings of the prevalence of undernutrition were also reported at national, urban and rural levels. For instance, Ethiopia had the lowest age-standardized mean BMI for both sexes, 16.8 kg/m^2^ for girls and 15.5 kg/m^2^ for boys [28]. BMI-for-age (thinness) in 2016, for non-pregnant girls of age 15–19 years was 5.7% at national level, 2.2% for urban dwellers and 6.3% for those with no or primary education [26]. Correspondingly, prevalence of thinness in boys (15–19) in 2016 was 28% at national, 22.9% for urban dwellers and 29.5% for those with no or primary education [26]. These might show that varying levels of prevalence of undernutrition existed in different localities, socioeconomic statuses, and demographic characteristics.

The findings in the current study were lower than global estimates of prevalence of thinness and overweight. Globally, underweight was persistently affecting children and adolescents of low-income countries, while overweight and obesity were rising across all ages, according to Global Nutrition Report [27]. By 2016, among school-age children and adolescents worldwide, 31.6% boys and 25.9% girls of age five–19 years old suffered from thinness/underweight [27]. Global prevalence of overweight was 19.2 and 17.5% among boys and girls, respectively [27].

Existing literature based prevalence of undernutrition on conventional measures of anthropometric indicators. This undermined the existing burden of malnutrition among school-age children and adolescents. In addition, the literature was scanty and fragmented into localities, age groups, sexes, and publication times. As a result, the evidence in it was too little and incomprehensive to fully understand the nutritional status of school-age children and adolescents.

Thus, the present study was the first to provide a more comprehensive measure of overall anthropometric failure that showed the magnitude of the prevalence of malnutrition among school-age children and adolescents in 50 randomly selected primary schools in all sub cities of Addis Ababa. It also presented individual conventional measures (stunting, thinness, and underweight) that could be used to compare with other studies.

The sex- and age-specific MUAC-for-age approach used to identify acute malnutrition, based on the reference group used in Mramba et al. [11], was, to our knowledge, also a first attempt of its kind. Our finding indicated that MUAC-for-age could identify extent of malnutrition in school-age children and adolescents closer to that indicated by the composite index (mCIAF). Similarly, it indicated an evident overall better classification of malnutrition which was in agreement with the finding that MUAC-for-age z-score was at least as effective as BMI-for-age z-score for assessing undernutrition among African children and adolescents of age 5–19 years [11].

The mixed effects regression model confirmed the observation that boys were more affected than girls by any level of malnutrition irrespective of the measurement applied; separate anthropometric indices, the composite index of anthropometric failure or MUAC-for-age (*p* < 0.0001) (Table 4). Similarly, it showed that stunting and thinness were more prevalent in early- and late-adolescent children than in those in middle childhood (p < 0.0001). This is in agreement with other reports which showed that prevalence of stunting and thinness in early adolescent girls (10 to 14 years old) was higher than that of girls of other age groups [29, 30]. The higher prevalence of underweight or overweight in adolescent boys than in adolescent girls, observed in our study, was also consistent with the global prevalence [27].

The variation in random effects across sub cities was significant (p < 0.0001) in the mixed effects regression fitted for any of the anthropometric measures. The variations in prevalence of malnutrition among sub cities, in terms of the conventional measures, MUAC-for-age and the modified composite index of anthropometric failure (mCIAF) might be due to the uneven and distribution of low-income households among the *kebeles* in the city [31, 32]. Variations were also reported in poverty indices of food consumption and calorie intake among the different sub cities of Addis Ababa [33].

Although this study showed the nutritional status of school-age children and adolescents as they first joined the SFP, subsequent study will further conduct repeated measurements at regular intervals to assess changes in nutritional status and academic performance of schoolchildren involved in this study.

## Conclusion

This study has shown that using mCIAF and sex-dependent MUAC-for-age give a better understanding of the extent of malnutrition among school-age children and adolescents which is undermined by measurements based on individual conventional indices. The large proportions of anthropometric failure and acute malnutrition indicated that children and adolescents were attending school under a high burden of hunger and malnutrition. Thus, improving the daily energy consumption through adequate amount and diversified nutritious food would revert malnutrition in school-age children and adolescents. The recently introduced school feeding program may benefit school-age children and adolescents to maintain proper physical growth.

## Data Availability

The datasets used and/or analyzed during the current study can be available from the corresponding author on reasonable request.

## Abbreviations

BMI: Body Mass Index
MAM: Moderate Acute Malnutrition
mCIAF: Modified Composite Index of Anthropometric Failure (mCIAF)
MUAC: Mid-Upper Arm Circumference
ROC: Receiver operating characteristic (ROC)
SAM: Severe Acute Malnutrition
